# Development of chloroplast transformation for five species in the genus *Nicotiana*

**DOI:** 10.1111/tpj.70542

**Published:** 2025-10-27

**Authors:** Qiuci Luo, Stephan Obst, Shengxin Chang, Stephanie Ruf, Ralph Bock

**Affiliations:** ^1^ Max‐Planck‐Institut für Molekulare Pflanzenphysiologie (MPI‐MP) Am Mühlenberg 1 D‐14476 Potsdam‐Golm Germany; ^2^ Present address: Henry Fok College of Biology and Agriculture Shaoguan University Shaoguan 512005 China

**Keywords:** plastid, chloroplast, plastid transformation, chloroplast transformation, Solanaceae, *Nicotiana benthamiana*, *Nicotiana glauca*, *Nicotiana langsdorffii*, *Nicotiana longiflora*, *Nicotiana otophora*, DsRed

## Abstract

Technologies for the stable genetic transformation of the plastid (chloroplast) genome are currently restricted to a small number of species. The development of highly efficient tissue culture, regeneration, and selection procedures represents the major hurdle that needs to be overcome to extend the species range of the transplastomic technology. Here, we report the development of efficient plastid transformation protocols for five species in the genus *Nicotiana*: the model species *N. benthamiana*, the tree tobacco *N. glauca*, the ornamental plants *N. langsdorffii* and *N. longiflora*, and the wild species *N. otophora*. We have optimized medium composition for efficient regeneration from leaf explants in all five species and determined suitable selection conditions for plastid transformation. We successfully isolated multiple transplastomic lines for each species and also generated lines that express the fluorescent reporter protein DsRed. Molecular and genetic analyses confirmed the homoplasmic state of the transplastomic lines and demonstrated maternal inheritance of the transgenes. Our work makes plastid genome engineering available for a set of new species and enables new applications in horticultural research and ecology. It also informs the future development of plastid transformation technology for other species.

## Significance Statement

In this work, robust methods for plastid genome engineering were developed for five species in the genus *Nicotiana*. The developed protocols will enable new applications in horticultural research and ecology, and inform the future development of plastid transformation technology for recalcitrant species.

## INTRODUCTION

Technologies for engineering the chloroplast genomes of plants by stable genetic transformation have provided a wealth of new fundamental insights into plant biology (Bock, 2015; Maliga, 2004). For example, they enabled mechanistic investigations into all steps in plastid gene expression (e.g., Allison et al., 1996; Allison & Maliga, 1995; Bock & Koop, 1997; Staub & Maliga, 1993), and made it possible to functionally analyze genes and open reading frames in the chloroplast genome (e.g., Hager et al., 2002; Ruf et al., 1997). They also facilitated novel approaches in experimental evolution (Fuentes et al., 2012; Huang et al., 2003; Stegemann et al., 2003; Stegemann & Bock, 2009), and enabled the study of a number of biological processes and phenomena that had not been amenable to rigorous experimentation before (e.g., Krämer et al., 2024; Ruf et al., 2007; Thyssen et al., 2012). Moreover, plastid genome engineering has opened up new possibilities in biotechnology and synthetic biology, and has been extensively applied in molecular farming (Bock & Warzecha, 2010; Tregoning et al., 2003), metabolic pathway engineering (Fuentes et al., 2018; Lu et al., 2013; Lu et al., 2017), and resistance engineering (De Cosa et al., 2001; McBride et al., 1995; Ye et al., 2001; Zhang et al., 2015).

Persuasive attractions of the chloroplast transformation technology include the high levels of transgene expression that often are achieved (De Cosa et al., 2001; Oey et al., 2009), the absence of transgene silencing mechanisms from plastids, the possibility to conveniently express multiple transgenes by stacking them into synthetic operons, and the increased transgene containment provided by the maternal mode of plastid inheritance in most crops (reviewed, e.g., in Maliga, 2004; Bock, 2015).

Although the plastid transformation technology clearly has had a transformative impact on both basic and applied research, its wider application is hampered by the very small number of species that can be transformed. About 35 years after the first report on stable chloroplast transformation in tobacco (*Nicotiana tabacum*; Svab et al., 1990), the list of transformable species is still very short and workable protocols are available for only a few species. This is not for lack of effort by the academic and the industrial sectors, but rather due to the strong dependence of successful chloroplast transformation on highly efficient regeneration and selection protocols that allow the isolation of chloroplast‐transformed (transplastomic) plants. Besides tobacco, in which the vast majority of research has been conducted, reliably transformable species currently include tomato (Apel & Bock, 2009; Ruf et al., 2001), potato (He et al., 2020; Sidorov et al., 1999; Valkov et al., 2011), lettuce (Harada et al., 2014; Kanamoto et al., 2006; Ruhlman et al., 2007), poplar (Okumura et al., 2006; Xu et al., 2020), petunia (Zubko et al., 2004) and the model plant *Arabidopsis thaliana* (Ruf et al., 2019; Ruf et al., 2025). Unfortunately, there is no universal plastid transformation protocol, and every new species to be transformed requires substantial efforts and individual optimization of the procedures involved in the selection and regeneration of transplastomic clones. By contrast, the DNA delivery process is largely universal and species‐independent, in that the particle gun‐mediated (biolistic) transformation method is capable of delivering foreign DNA into the cells of essentially all plant species.

Here we report the development of efficient plastid transformation protocols for five species in the genus *Nicotiana*. By screening a large number of species in the genus for their tissue culture properties, we identified a subset of species that displayed favorable properties and showed high regeneration rates on optimized culture media. We constructed species‐specific plastid transformation vectors and succeeded with the generation of stably transformed lines at high frequency in all five species. The selected transplastomic events could be regenerated into fertile plants and showed maternal transmission of the plastid transgenes into the next generation. By making ornamental plants, a tree, and a wild species amenable to engineering of the plastid genome, our work extends the species range of the technology to plants that can serve as new models for horticultural and forestry research and studies of plant ecology.

## RESULTS

### Optimization of tissue culture and plant regeneration for *Nicotiana* species

**Figure 1:**
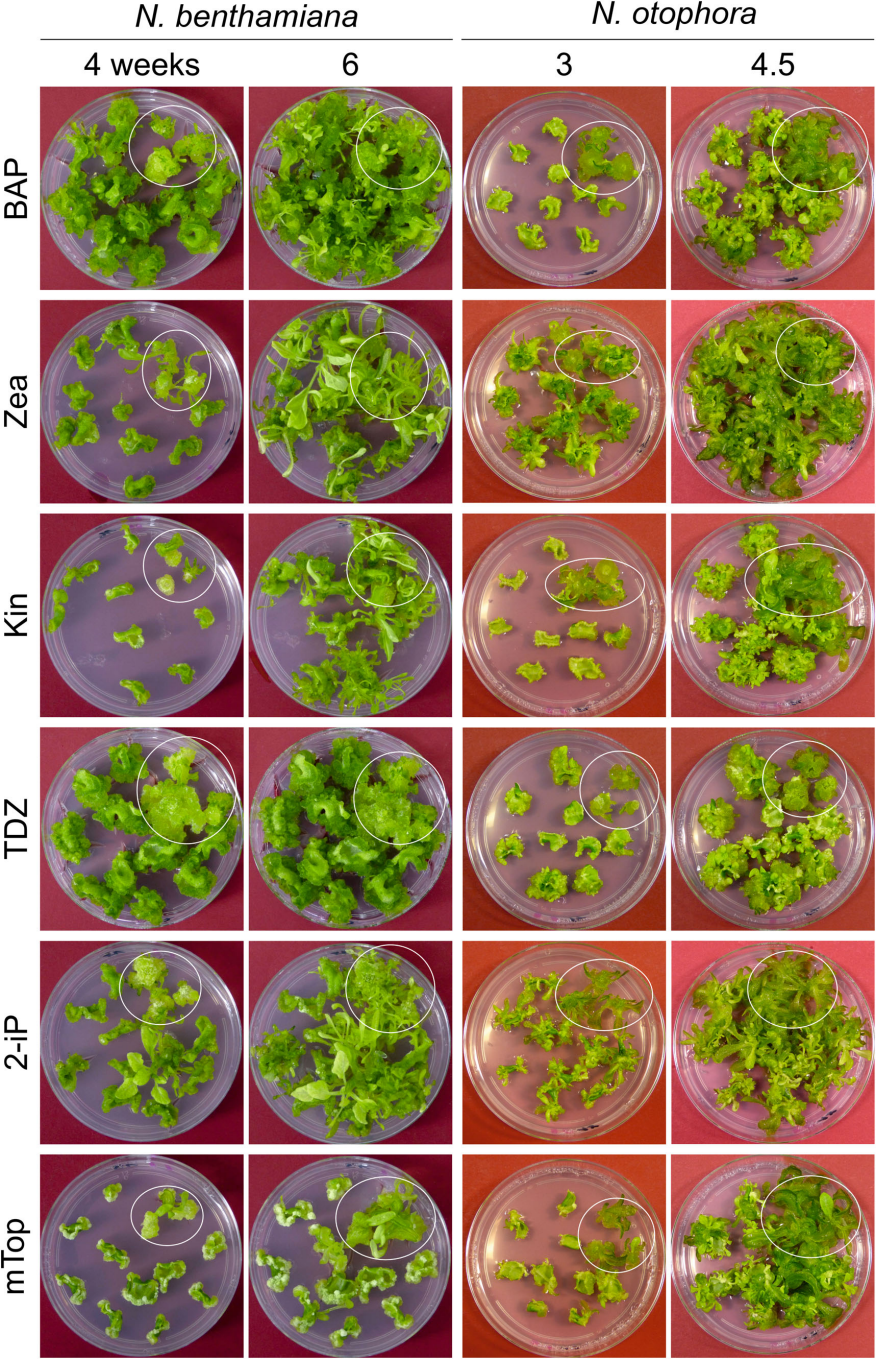
Evaluation of the regeneration capacities of explants from *N. benthamiana* and *N. otophora*. Regeneration media with six different cytokinins were tested in this experiment, and regeneration was documented at two time points. Stem discs tested for regeneration are located within the white circle in the upper right corner of the Petri dishes. All other explants were from leaves. BAP: 6‐benzylaminopurine; Kin: kinetin; mTOP: meta‐topolin; TDZ: thidiazuron; Zea: zeatin; 2‐iP: N6‐2‐isopentenyladenine. For the other 15 species tested, see Figures S1–S4.

The allotetraploid species *Nicotiana tabacum* (Ntab) is routinely used for chloroplast transformation experiments due to its highly efficient and fast regeneration in tissue culture (Bock, 2015; Svab & Maliga, 1993). In the genus *Nicotiana*, chloroplast transformation protocols were published also for *Nicotiana sylvestris* (Nsyl; Maliga & Svab, 2011) and *Nicotiana benthamiana* (Nbent; Chen et al., 2014), but have only rarely been used. To assess the amenability of species in the genus to plastid transformation, we conducted systematic tests for regeneration efficiency in 17 *Nicotiana* species (Figure 1; Table 1; Figures S1–S4). When testing different media and phytohormones, we noticed that especially the type of cytokinin had a substantial impact on shoot regeneration from leaf and stem explants, and in different species, different cytokinins conferred optimal regeneration (Figure 1; Table 1; Figures S1–S4). In these experiments, we identified five species, *N. benthamiana*, *N. glauca* (Nglau), *N. langsdorffii* (Nlangs), *N. longiflora* (Nlong), and *N. otophora* (Noto), that displayed good shoot regeneration on optimized culture media (Figure 1; Table 1). Overall, the regeneration capacity of these five species was judged to be high enough to proceed with the establishment of chloroplast transformation protocols.

**Table 1:** Assessment of the shoot regeneration capacity of leaf and stem explants from 17 *Nicotiana* species

Species	BAP		Zea		Kin		TDZ		2‐iP		mTop	
	Leaf piece	Stem disc	Leaf piece	Stem disc	Leaf piece	Stem disc	Leaf piece	Stem disc	Leaf piece	Stem disc	Leaf piece	Stem disc
Nacua	0	0	0	0	0	0	0	0	0	0	0	0
Nala	1	—	1	—	1	—	0	—	0	—	1	—
Natt	0	0	0	0	0	0	0	0	0	0	0	0
Nbent	3	2	2	2	3	3	1	1	3	3	0	1
Nglau	3	3	3	3	4	4	3	4	3	3	1	2
Nglut	2	2	2	1	1	1	2	1	1	1	0	1
Nkaw	0	1	0	1	0	1	0	1	0	1	0	1
Nlangs	1	—	1	—	3	—	2	—	1	—	2	—
Nlong	2	—	2	—	3	—	2	—	1	—	2	—
Nnoc	0	0	0	0	0	0	0	0	0	0	0	0
Nobt	0	—	0	—	0	—	0	—	0	—	0	—
Noto	4	3	4	3	4	3	2	1	3	3	3	3
Npan	0	1	0	1	0	0	0	1	0	1	0	0
Nsyl	4	—	4	—	4	—	4	—	4	—	4	—
Ntab	4	3	4	3	4	4	4	3	2	3	2	3
Ntofo	0	1	0	1	0	2	0	1	0	1	0	—
Nund	0	2	1	2	0	2	1	3	1	3	0	3

Regeneration ability was quantified on a scale from 0 to 4, with 0 indicating no capacity for shoot differentiation and 4 representing the highest level of shoot regeneration observed across all species.

—, not tested; 2‐iP, N6‐2‐isopentenyladenine; BAP, 6‐benzylaminopurine; Kin, kinetin; mTOP, meta‐topolin; TDZ, thidiazuron; Zea, zeatin.

Chimeric *aadA* genes that confer resistance to the aminoglycoside antibiotic spectinomycin represent the most efficient selectable marker gene for stable transformation of the plastid genome (Bock, 2015; Svab & Maliga, 1993). We, therefore, examined the sensitivity of the five chosen *Nicotiana* species to spectinomycin. While Nbent, Nglau, Nlangs, and Noto showed similar sensitivity to the antibiotic as Ntab in that leaf pieces completely bleached out on regeneration medium containing 500 mg L^−1^ spectinomycin, Nlong leaf tissue exhibited an unexpectedly high tolerance to spectinomycin. In the presence of 500 mg L^−1^ spectinomycin, leaf explants largely retained their photosynthetic pigments and displayed extensive callus growth along the leaf margins (Figure S5). Nlong also tolerated high concentrations of streptomycin (Figure S5), another aminoglycoside antibiotic to which the *aadA* gene confers resistance (Bock, 2001; Svab & Maliga, 1993). When Nlong explants were tested for sensitivity to elevated concentrations of both drugs, we found that concentrations of 1500 mg L^−1^ spectinomycin or 1000 mg L^−1^ streptomycin were sufficient to effectively inhibit cell division and suppress callus growth (Figure S5).

### Construction of chloroplast transformation vectors

**Figure 2:**
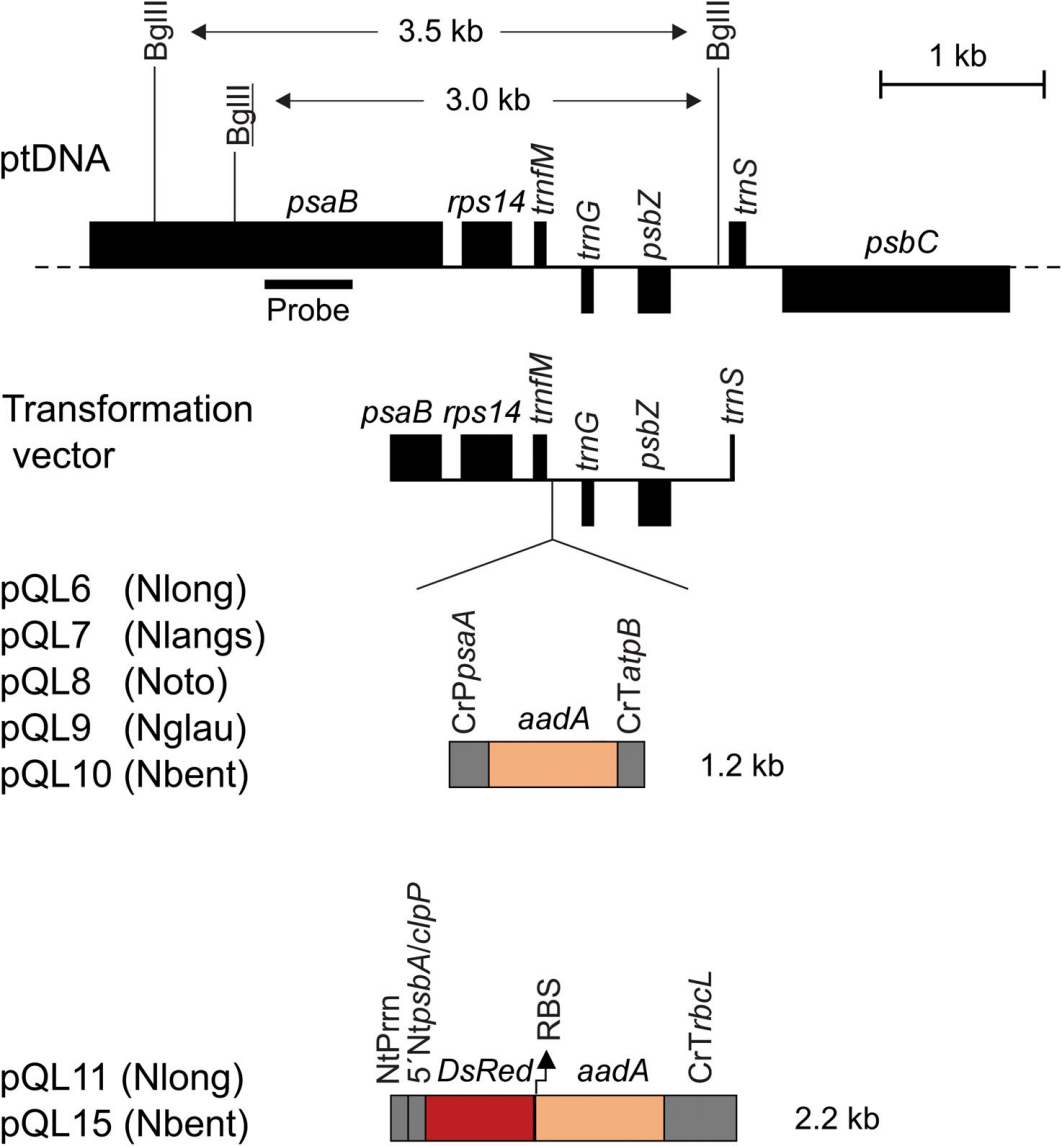
Physical map of the targeting region in the plastid genomes (ptDNA) of the five *Nicotiana* species selected for the development of plastid transformation and construction of transformation vectors. Filled black boxes represent plastid genes. For genes above the line, the direction of transcription is from left to right, and for genes below the line, the direction of transcription is from right to left. The genomic region shown was cloned from the five species to facilitate transgene integration into the plastid genome by homologous recombination. Transgenes were targeted to the intergenic spacer between the *trnfM* and *trnG* genes. Vectors pQL6, pQL7, pQL8, pQL9, and pQL10, each harboring an *aadA* cassette conferring resistance to spectinomycin, were utilized for chloroplast transformation in Nlong, Nlangs, Noto, Nglau, and Nbent, respectively. Vectors pQL11 and pQL15 were designed for Nlong and Nbent and additionally express the red fluorescent protein DsRed from a dicistronic transcript (Hertle et al., 2021). The BglII restriction sites indicated in the physical map of the ptDNA were used for Southern blot analysis, and the binding site of the hybridization probe is indicated as a black horizontal bar. It should be noted that Noto has two BglII restriction sites in the *psaB* gene, whereas the other species have only one. The Noto‐specific BglII site (underlined) results in a restriction fragment that is 0.5 kb smaller than that of the other species (3.0 kb instead of 3.5 kb). CrP*psaA*: promoter of the plastid *psaA* gene from *Chlamydomonas reinhardtii*; CrT*atpB*: 3′ UTR of the plastid *atpB* gene from *C. reinhardtii*; NtP*rrn*: rRNA operon promoter from *N. tabacum*; 5′Nt*psbA*/*clpP*: chimeric 5′ UTR from the chloroplast *psbA* and *clpP* genes of *N. tabacum*; RBS: synthetic Shine‐Dalgarno sequence (ribosome binding site); CrT*rbcL*: 3′ UTR from the chloroplast *rbcL* gene of *C. reinhardtii*; *aadA*: spectinomycin‐resistant gene; *DsRed*: gene encoding a red fluorescent protein. The sizes of the two transgene cassettes are given in kilobases (kb).

Transgene integration into the plastid genome occurs exclusively by homologous recombination. A commonly used insertion site resides in the *psaB/trnS* region of the chloroplast genome, between the *trnfM* and *trnG* genes (Ruf et al., 2001; Ruf et al., 2019; Figure 2). To assess the sequence conservation of the *psaB/trnS* region, we compared the corresponding genomic sequences of the five *Nicotiana* species. While chloroplast genome sequences were available for *N. tabacum*, *N. benthamiana*, *N. glauca*, and *N. otophora* (Asaf et al., 2016; Stegemann et al., 2012; Yukawa et al., 2005), no sequence information was available for *N. langsdorffii* and *N. longiflora*. We, therefore, sequenced the complete chloroplast genomes of the two species (see Methods; accession numbers: PV995076 and PV995077), and subsequently aligned the sequences of the *psaB/trnS* regions of all five species with that of the reference sequence from Ntab (Figure S6). In general, the sequence of this region is well conserved between species of the genus. However, a number of single‐nucleotide polymorphisms (SNPs) and small insertions and/or deletions (InDels) were identified (Figure S6). Although the presence of a few SNPs in flanking regions of plastid transformation vectors usually does not substantially reduce transformation efficiency, sequence deviations between vector and resident plastid genome can cause resolution of Holiday junctions and result in complex recombination patterns (Kavanagh et al., 1999). We, therefore, decided to construct species‐specific transformation vectors that are based on the flanking genomic sequences from each of the five target species (Figure 2). In this way, five plastid transformation vectors were constructed that harbor the selectable marker gene *aadA* flanked by species‐specific chloroplast sequences (vectors pQL6, pQL7, pQL8, pQL9, and pQL10; Figure 2).

To be able to visually assess successful chloroplast transformation, we additionally constructed two vectors that harbor the *DsRed* gene. *DsRed* encodes a red fluorescent protein that can serve as a reporter of plastid transgene expression (Hertle et al., 2021). In plastid transformation vectors pQL11 and pQL15 (Figure 2), the DsRed protein is co‐expressed with the *aadA* coding region from a dicistronic transcript, in which each of the two coding regions has its own ribosome binding site (Hertle et al., 2021). Vectors pQL11 and pQL15 were used for additional sets of plastid transformation experiments in Nlong and Nbent, respectively.

### Generation of stable transplastomic lines for the five *Nicotiana* species

**Figure 3:**
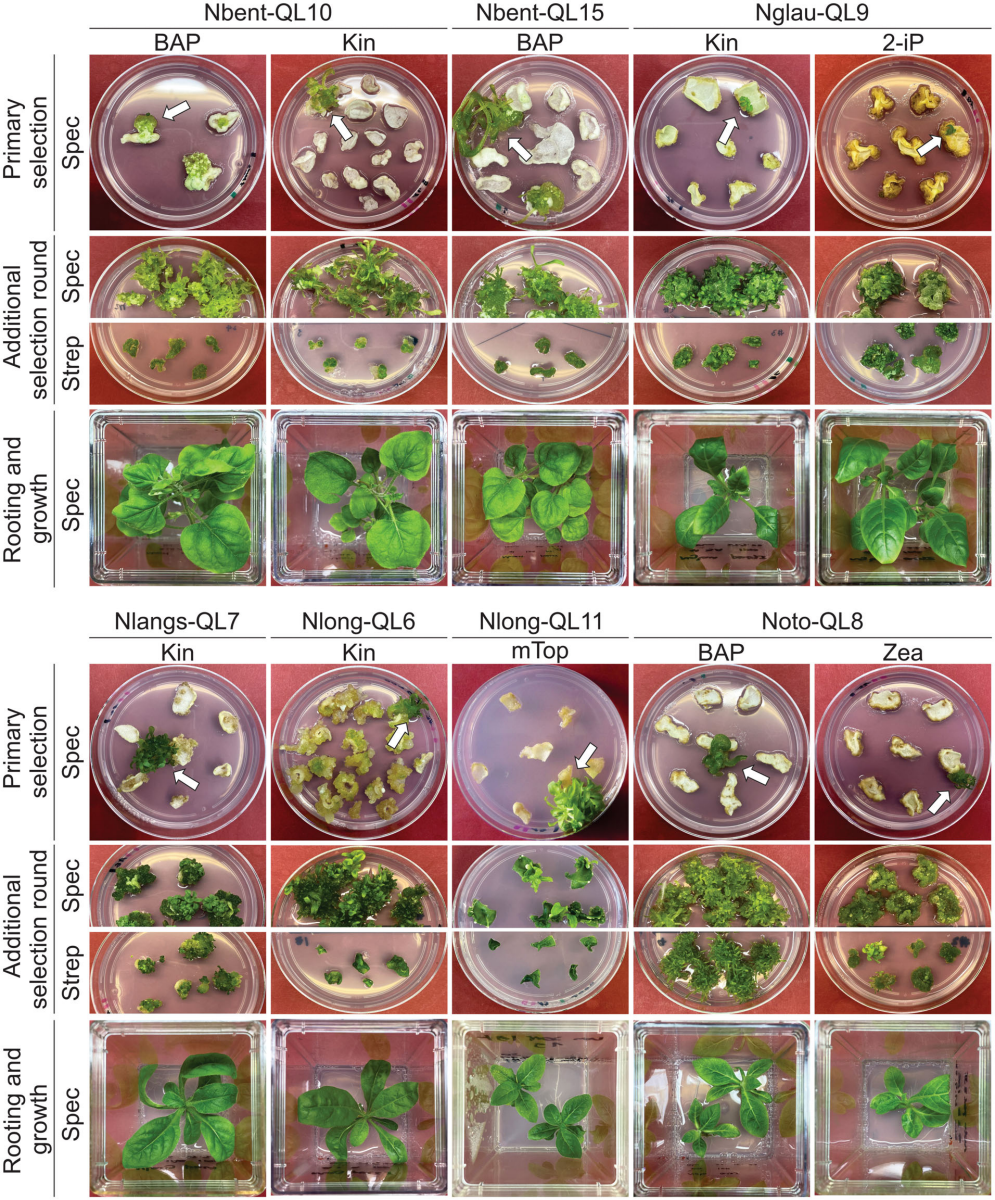
Generation of transplastomic plants for five species in the genus *Nicotiana*. The figure illustrates the selection process for transplastomic plants resistant to spectinomycin and streptomycin in the five species and encompasses ten independent transformation experiments. The cytokinin used in the regeneration medium for each transformation experiment is given below the experiment identifier (‘species’–‘transformation vector’). In each experiment, the same regeneration medium was used for primary selection and the additional selection rounds. In primary selection, spectinomycin‐resistant lines were identified (white arrows). The same lines are shown as examples in the additional round of selection, in which tissue explants were separately tested for resistance to spectinomycin and streptomycin. Regenerants from additional selection cycles on spectinomycin were tested for homoplasmy by RFLP analyses (Figure 4; Figure S7). Finally, putative homoplasmic transplastomic shoots were rooted in hormone‐free medium containing spectinomycin for transfer to soil and further cultivation in the greenhouse.

Having optimized regeneration and selection conditions, and having constructed a set of species‐specific plastid transformation vectors, we next conducted particle gun‐mediated (biolistic) transformation experiments in all five species (Figure 3; Table 2). In view of the high tolerance of Nlong to the selection agent spectinomycin, different selection schemes were tested for this species. They involved either direct selection on elevated antibiotic concentrations, or alternatively, initial selection on 500 mg L^−1^ spectinomycin followed by transfer to fresh medium containing elevated antibiotic concentrations (1000 or 2000 mg L^−1^; Table 2). For all species, in which two or more cytokinins conferred similarly high regeneration efficiencies, we also conducted selection experiments in the presence of different cytokinins (Figure 3; Table 2).

**Table 2:** Summary of plastid transformation experiments conducted in five *Nicotiana* species

Species	Vector	Auxin in SIM (0.1 mg L^−1^)	Cytokinin in SIM (mg L^−1^)	Selection agent: Spectinomycin (mg L^−1^)	Number of shots^b^	Selected leaf pieces^e^	Medium change (weeks)	Appearance of resistant lines (weeks)	Number of primary resistant lines	Number of primary resistant lines with shoot regeneration	Number of analyzed lines for streptomycin resistance^f^	Streptomycin sensitive lines^f^	Streptomycin‐resistant lines^f^	Streptomycin‐resistant lines tested by RFLP^g^	Streptomycin‐resistant lines confirmed by RFLP
Nbent	pQL10	NAA	1.0 BAP	500	1	322	6–7	13–26	46	24	24	6	18	6	6
Nbent	pQL10	IAA	2.0 Kin	500	1	350	5	16–31	17	17	17	8	9	6	6
Nbent	pQL15	NAA	1.0 BAP	500	1	420	6–7	12–20	114	12	12 + (78)^f^	5 + (4)^f^	7 + (74)^f^	5	5
Nglau	pQL9	IAA	2.0 Kin	500	1	392	6–7	7–12	11	11	11	1	10	6	6
Nglau	pQL9	IAA	2.0 2‐iP	500	1	280	6–7	19–25	2	2	2	—	2	1	1
Nlangs	pQL7	IAA	2.0 Kin	500	2	791	5	10–25	20	18	18	—	18	7	7
Nlong	pQL6	IAA	2.0 Kin	500 → 1000/2000^a^	1	420	5	10–30	15	15	15	6	9	6	6
Nlong	pQL6	IAA	0.5 mTop	500 → 1000/2000^a^	1	350	5	10–18	5	5	5	5	—	na	na
Nlong	pQL11	IAA	2.0 Kin	1000	0.5^c^	168	7–8	24–46	3	3	3	3	—	na	na
Nlong	pQL11	IAA	2.0 Kin	2000	0.5^c^	168	5	11–28	12	12	12	10	2	2	2
Nlong	pQL11	IAA	0.5 mTop	1000	0.5^d^	168	5	—	—	—	—	na	na	na	na
Nlong	pQL11	IAA	0.5 mTop	2000	0.5^d^	168	5	11–21	7	7	7	6	1	1	1
Noto	pQL8	NAA	1.0 BAP	500	1	322	6–7	13–26	35	14	14	1	13	7	7
Noto	pQL8	IAA	2.0 Zea	500	1	350	5	10–19	13	10	10	4	6	5	5

—, not obtained; na, not applicable.

^a^ Initial selection was performed for 5 weeks using 500 mg L^−1^ spectinomycin, followed by a subsequent increase to either 1000 mg L^−1^ or 2000 mg L^−1^.

^b^ Using the hepta adaptor and bombarding a Petri dish fully covered with leaves.

^c^ Were from the same bombarded Petri dish, with the plant material split into selection at spectinomycin 1000 or 2000 mg L^−1^.

^d^ Were from the same bombarded Petri dish, with the plant material split into selection at spectinomycin 1000 or 2000 mg L^−1^.

^e^ 5 × 5 mm leaf pieces.

^f^ Most lines used for streptomycin resistance analysis had already regenerated shoots at the time of testing, and explants from shoots were used for the analysis. The only exception was Nbent transformed with pQL15, where some lines had remained at the callus stage and were tested as callus samples (numbers shown in parentheses).

^g^ Not all streptomycin‐resistant lines underwent RFLP analysis; only RFLP‐positive lines were transferred to the greenhouse for seed production.

Spectionomycin‐resistant lines were obtained for all species and with all vectors (Figure 3; Table 2). Since spectinomycin resistance can also arise spontaneously through specific point mutations in the plastid 16S rRNA gene (Svab & Maliga, 1991), additional resistance tests were performed by exposing tissue explants from spectinomycin‐resistant clones to streptomycin. While the *aadA* marker gene confers resistance to both aminoglycosides, the point mutations in the 16S rRNA gene are strictly specific to spectinomycin and do not confer resistance to streptomycin (Bock, 2001). The streptomycin tests eliminated a number of spontaneous mutants and identified multiple resistant lines in each species as strong candidates for being true transplastomic events (Table 2).

The candidate lines were subjected to an additional round of selection and regeneration in the presence of spectinomycin (Figure 3) to eliminate residual wild‐type (WT) copies of the highly polyploid chloroplast genome (Greiner et al., 2020), and isolate homoplasmic transplastomic lines that contain a homogeneous population of transformed plastid genome molecules.

**Figure 4:**
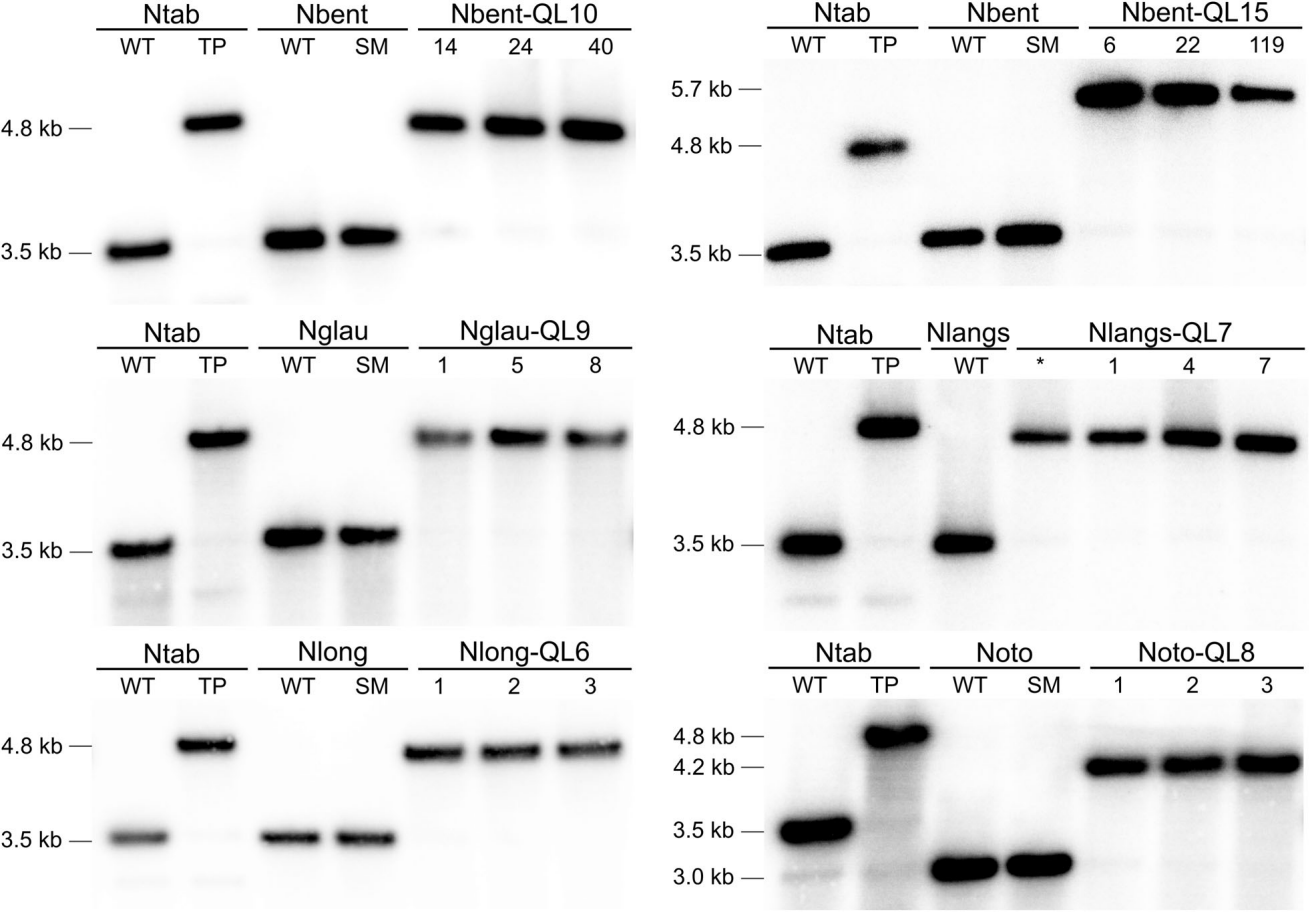
RFLP analysis of transplastomic *Nicotiana* lines by DNA gel blot analyses. Total DNA was extracted from shoots undergoing rooting after the additional selection round (Figure 3). DNA samples were digested with the restriction endonuclease BglII, and the resulting fragments were separated by agarose gel electrophoresis and hybridized to a radiolabeled probe (cf. Figure 2). Three independent transplastomic lines were included for each species and transformation construct (with the line numbers given below the horizontal lines). The sizes of the hybridizing fragments are given in kb at the left. *: a line that preliminarily had been identified as a spontaneous mutant based on apparent sensitivity to streptomycin, but was shown to be transplastomic by Southern blotting; WT: wild‐type; TP: transplastomic plant; SM: spontaneous spectinomycin‐resistant mutant. For additional Southern blot data, see Figure S7.

To ultimately confirm the transplastomic status of the doubly antibiotic‐resistant lines and assess homoplasmy, restriction fragment length polymorphism (RFLP) analyses by Southern blotting were conducted (Figure 4 and Figure S7). These experiments revealed the expected size shift in the restriction fragment harboring the transgene(s) and the complete, or nearly complete, absence of the hybridization signal for the WT genome. In a few cases, very faint WT‐like hybridization signals were detected in the transplastomic lines upon strong exposure of the blots (Figure 4 and Figure S7). According to previous studies, these signals do not come from the presence of residual WT copies of the plastid genome, but instead, originate from so‐called promiscuous plastid DNA: chloroplast DNA sequences that have integrated into the nuclear genome due to intracellular gene transfer (also referred to as endosymbiotic gene transfer; Hager et al., 1999; Ruf et al., 2000; Bock, 2017).

To be able to ultimately confirm the homoplasmic status of the transplastomic lines by inheritance assays (Krech et al., 2012; see below), putative homoplasmic plantlets were rooted on phytohormone‐free culture media (Figure 3), transferred to soil, and grown to maturity under standard greenhouse conditions.

### Maternal inheritance of chloroplast transgenes

**Figure 5:**
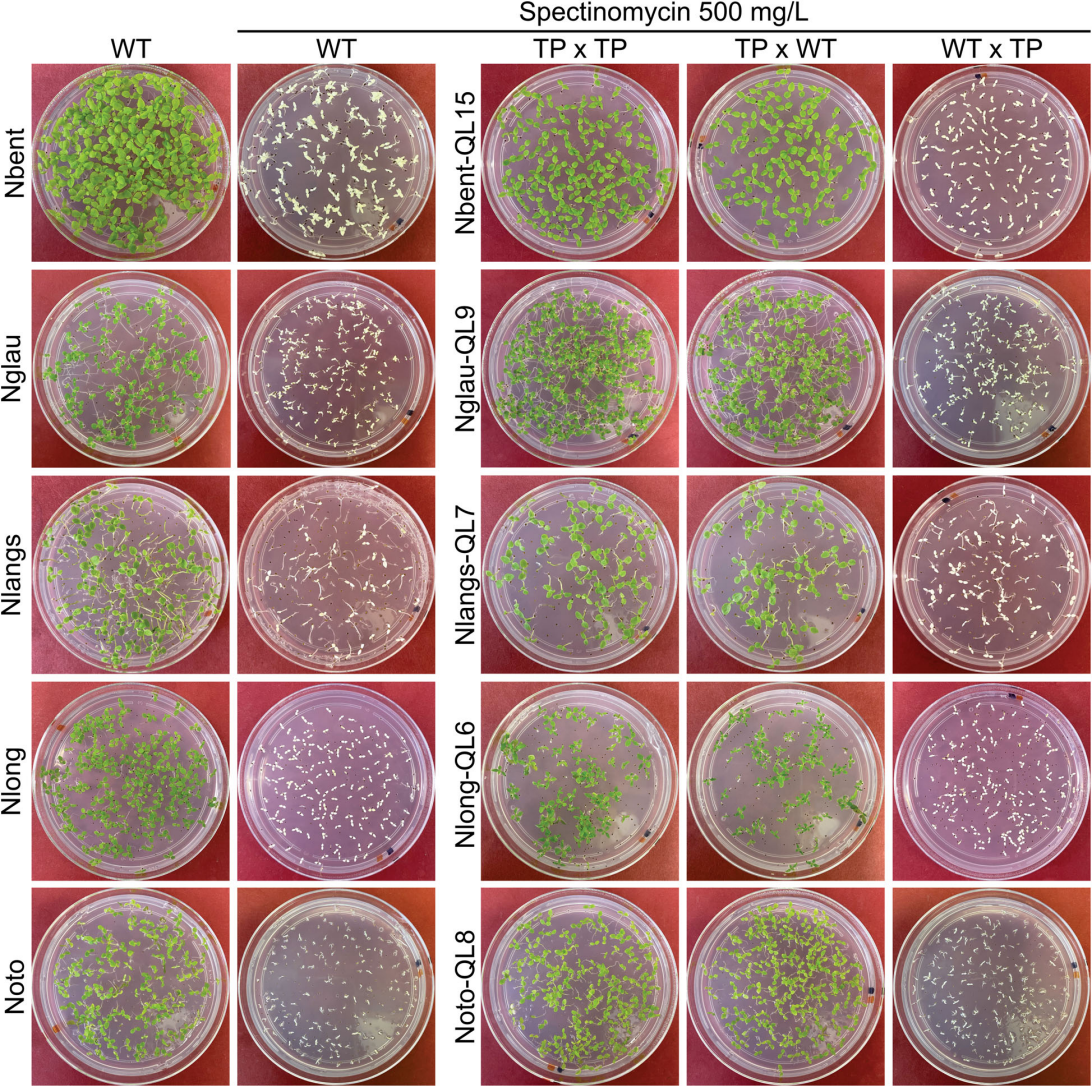
Seedling resistance tests to confirm homoplasmy and maternal inheritance of the plastid transgene conferring spectinomycin resistance. One transplastomic line for each species is exemplarily shown. Surface‐sterilized wild‐type (WT) seeds and seeds obtained from crosses with transplastomic plants were sown on synthetic medium without (left) or with 500 mg L^−1^ spectinomycin. Seeds derived from self‐pollinated transplastomic plants (TP × TP), transplastomic plants fertilized with wild‐type pollen (TP × WT), and reciprocal crosses, in which the transplastomic plant served as pollen donor for the wild‐type (WT × TP), were assayed, and the inheritance of the spectinomycin resistance was analyzed.

When greenhouse‐grown transplastomic plants flowered, individual flowers were self‐pollinated, and reciprocal crosses with WT plants were conducted. The progenies of all crosses were then assayed for inheritance of the spectinomycin resistance trait by germinating seeds on synthetic medium in the presence of the antibiotic. The offspring from all self‐pollinated transplastomic flowers were uniformly resistant to spectinomycin (Figure 5), consistent with the maternal inheritance of chloroplast transgenes (Chung et al., 2023) and the homoplasmic status of the transplastomic lines.

In line with the uniparental transmission of plastids and their genomes, crosses with transplastomic plants as maternal parent yielded progeny that were homogeneously resistant to spectinomycin. Conversely, crosses in which the transplastomic plant served as paternal parent produced only antibiotic‐sensitive progeny (Figure 5), due to exclusion of paternal organelles from sexual transmission.

Together, these data confirmed the plastid encoding of the spectinomycin resistance trait and firmly established the homoplasmy of all transplastomic lines generated for the five species.

### Phenotypes of transplastomic lines and DsRed reporter expression

**Figure 6:**
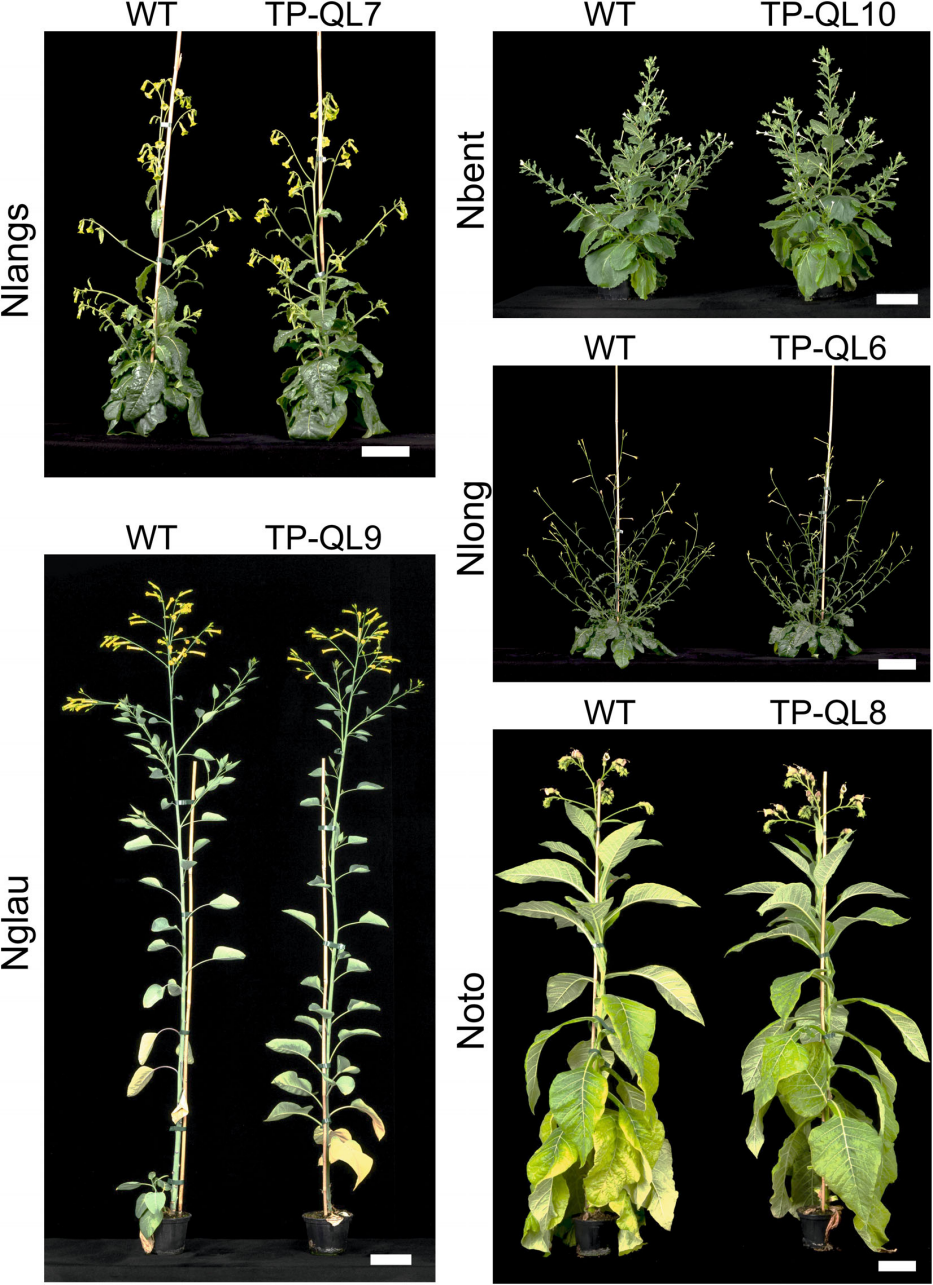
Phenotypes of transplastomic plants grown in the greenhouse. Transplastomic plants of each *Nicotiana* species were raised from seeds and grown under greenhouse conditions along with wild‐type (WT) control plants. Scale bars: 10 cm.

Transplastomic plants obtained for all five species grew normally in the greenhouse and were indistinguishable from WT plants (Figure 6). Also, flowering time, fertility, and seed set were comparable to the WT control. These observations are consistent with a large number of previous studies that expressed the *aadA* selectable marker gene and genes for fluorescent reporter proteins from the same insertion site in the chloroplast genome of the model plant *Nicotiana tabacum* (e.g., Emadpour et al., 2015; Ruf et al., 2007).

**Figure 7:**
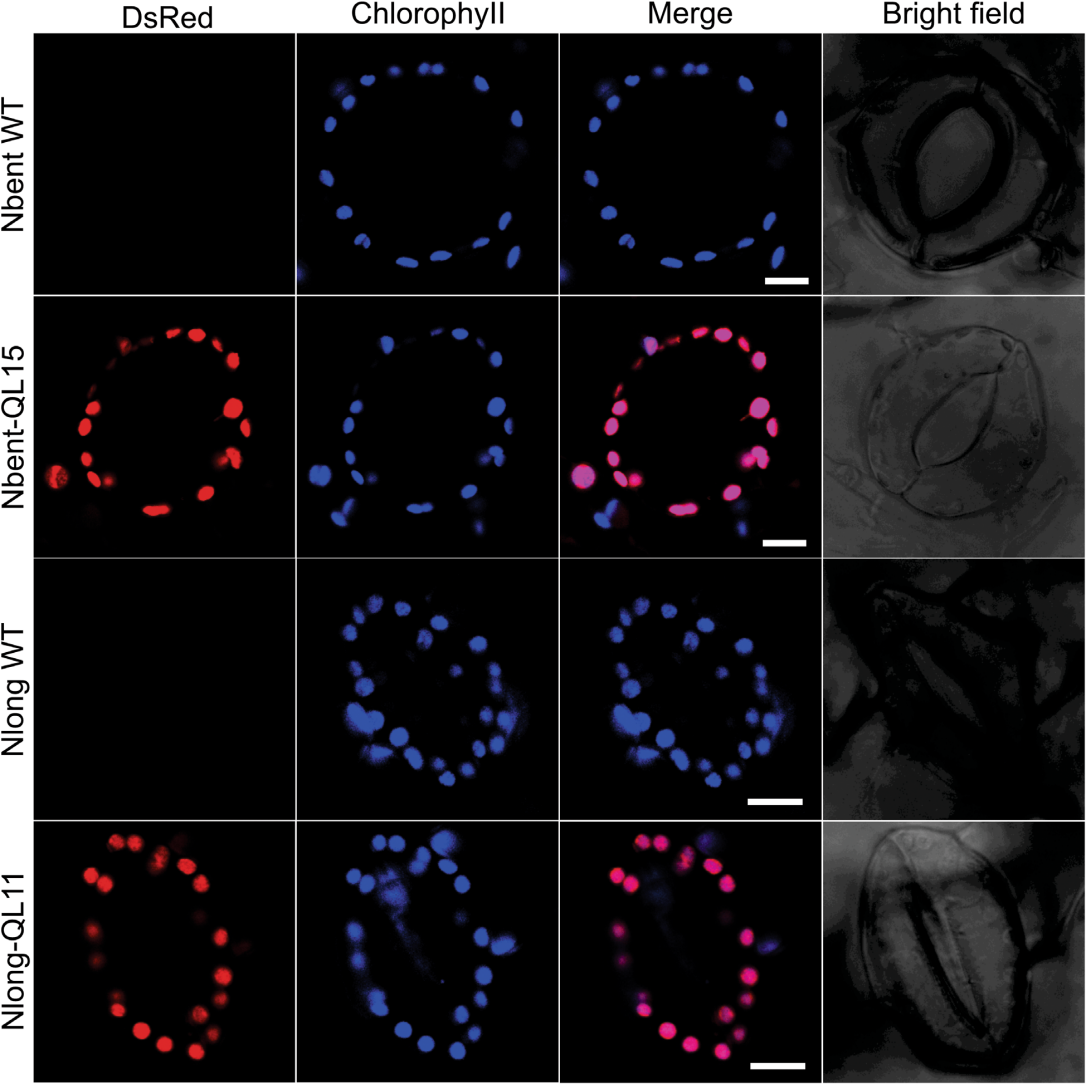
Detection of chloroplast DsRed expression in leaf epidermal guard cells of transplastomic Nbent‐QL15 and Nlong‐QL11 plants by confocal laser‐scanning microscopy. DsRed fluorescence is shown in red, and chlorophyll fluorescence in blue. The two wild‐types (Nbent WT, Nlong WT) are included for comparison. The merged images show the overlap of chlorophyll and DsRed fluorescences. Scale bars: 10 μm.

The successful generation of transplastomic Nlong and Nbent plants with vectors pQL11 and pQL15 (that additionally carry a *DsRed* cassette; Figure 2) allowed us to also analyze the subcellular localization of the DsRed fluorescence and confirm its localization within the chloroplast compartment (Figure 7). The presence of bright DsRed fluorescence within the chloroplasts (and the absence of red fluorescence from the WT control; Figure 7) confirmed high‐level expression of the reporter protein and its confinement to the chloroplast compartment.

## DISCUSSION

Our work reported here provides protocols for plastid genome engineering in five species from the genus *Nicotiana*, four of which have not been transformed before. Among the newly transformed species are two ornamental plants, a woody plant, and a wild species, all of which are now available for studies that either require chloroplast genome engineering or can benefit from it. Importantly, four of the species are diploid and thus more amenable to genetic approaches than the allotetraploid model plant *Nicotiana tabacum*, in which the vast majority of the previous work on chloroplast genome engineering has been conducted.

The key to success with the establishment of workable plastid transformation protocols for the five species has been (i) their identification in a systematic analysis of the regeneration properties of many species in the genus (Table 1), and (ii) the optimization of the tissue culture media and selection conditions (Figure 1; Figures S1–S5). The identification of the most suitable cytokinin for each species (by testing six different commercially available compounds) turned out to be particularly important, in that the different cytokinins had a strong effect on the efficiency of shoot regeneration in *in vitro* culture. Another interesting observation made in the course of this work was that, even within the same genus, species can display considerable differences in their sensitivity to spectinomycin, the selection agent most commonly used for chloroplast transformation (Figure S5; Table 2). The unusually high spectinomycin tolerance and undesired background regeneration observed in *N. longiflora* could be overcome by increasing the concentration of the antibiotic upon selection for transplastomic events (Table 2; Figure 3). These results illustrate that the careful determination of the suitable selection window of the antibiotic is a particularly important step in the optimization of the procedures involved in plastid transformation. Overall, our findings underscore the previous notion that the development of efficient protocols for the selection of transplastomic events and the regeneration of fertile homoplasmic lines is crucial to the establishment of chloroplast transformation technology in any new species.

The transformation frequencies obtained in our present study are sufficiently high to enable routine use of the technology in all five species. In a typical experiment, multiple transplastomic events are selected from a single shot (using the hepta adaptor setup of the helium‐driven particle gun; Table 2).

The five species successfully transformed in this study displayed relatively favorable tissue culture and regeneration properties (Figure 1; Figures S1–S4; Table 1) that could be optimized to a point at which chloroplast transformation became feasible. A number of other species in the genus showed considerable recalcitrance to efficient regeneration in tissue culture using the media and conditions tested in this work (Figures S1–S4; Table 1). Overcoming this recalcitrance will likely require different approaches, including systematic tests of various source tissues and/or genetic engineering approaches that improve the responsiveness of these species to tissue culture conditions (Ruf et al., 2019). Nonetheless, our work reported here (i) extends the species range of plastid transformation technology, (ii) makes species amenable to chloroplast genome engineering that can serve as models for horticultural research, forestry research, and ecological studies, and (iii) highlights strategies for the successful development of plastid transformation in other species.

## EXPERIMENTAL PROCEDURES

### Plant material and growth conditions

WT plants of a panel of 17 *Nicotiana* species were included in this study: *N. acuminata* (Nacua), *N. alata* (Nala), *N. attenuata* (Natt), *N. benthamiana* (Nbent), *N. glauca* (Nglau), *N. glutinosa* (Nglut), *N. kawakamii* (Nkaw), *N. langsdorffii* (Nlangs), *N. longiflora* (Nlong), *N. noctiflora* (Nnoc), *N. obtusifolia* (Nobt), *N. otophora* (Noto), *N. paniculata* (Npan), *N. sylvestris* (Nsyl), *N. tabacum* (Ntab), *N. tomentosiformis* (Ntofo), and *N. undulata* (Nund). All species are diploid with a chromosome number of 2n = 24, except for Nala and Nlangs with 2n = 18, Nlong with 2n = 20, Nbent with 2n = 38, and the allotetraploid species Ntab with 2n = 48 (Goodspeed, 1954). With the exception of Ntab and Nbent, seeds of all other species were obtained from the seed bank at IPK Gatersleben, Germany (https://www.ipk‐gatersleben.de/en/research/genebank).

Germination of surface‐sterilized seeds and seedling growth under aseptic conditions was performed in controlled environment facilities, with a 16‐h photoperiod at a light intensity of 55 μmol photons m^−2^ s^−1^ and a temperature of 25°C, followed by an 8‐h dark period at 20°C. Regeneration experiments were conducted under similar conditions but with a reduced light intensity of 25 μmol photons m^−2^ s^−1^. Plant growth in soil and seed production was carried out under standard greenhouse conditions (daylength: 16 h; day temperature: 25°C; night temperature: 20°C; average light intensity: 350 μmol photons m^−2^ s^−1^).

### Regeneration assays with different *Nicotiana* species

Sterile seeds of the 17 *Nicotiana* species were sown on synthetic medium containing 2% sucrose (MSsuc2; Murashige & Skoog, 1962). After germination, seedlings were individually transferred to Magenta boxes containing the same MSsuc2 medium for further growth.

For assessment of regeneration capacity, leaf explants from the first three fully expanded young leaves (approximately 5 × 5 mm in size) and stem discs (approximately 1.5 mm thick) were excised from plants grown in Magenta boxes for 3–5 weeks. The explants were placed onto six different shoot induction media (SIMs), each containing a specific cytokinin and the same concentration of the auxin indole‐3‐acetic acid (IAA; 0.1 mg L^−1^). The cytokinins used were: 6‐benzylaminopurine (BAP) at 3 mg L^−1^, zeatin (Zea) at 2 mg L^−1^, kinetin (Kin) at 2 mg L^−1^, thidiazuron (TDZ) at 0.1 mg L^−1^, N6‐2‐isopentenyladenine (2‐iP) at 2 mg L^−1^, and meta‐topolin (mTop) at 0.5 mg L^−1^. All SIMs are based on MSsuc2 and were supplemented with auxin and cytokinin. During the regeneration assays, no medium change was conducted, with the exception of Nlangs, where the medium was replaced after 5 weeks.

### Chloroplast genome sequencing and comparison of *Nicotiana* species

Chloroplast genome sequencing of *N. langsdorffii* and *N. longiflora* was conducted using the NovaSeq 6000 platform, employing the NovaSeq 6000 S2 Reagent kit for library construction (MPI‐MG Sequencing Facility, Berlin) from purified chloroplast DNA. Between 2.9 and 4.8 Gbp of sequence information was obtained for each species from paired‐end 2 × 100 bp reads. The sequencing output was then subjected to quality control and adapter trimming, utilizing TrimGalore v0.6.10 (https://github.com/FelixKrueger/TrimGalore) with the parameters ‐‐phred33 ‐‐quality 20. Contigs were assembled using GetOrganelle 1.7.5 (https://github.com/Kinggerm/GetOrganelle) with the parameters ‐R 20 ‐k 21,45,65,85,105. Sequence evaluation and construction of alignments were performed with the GeSeq package (Tillich et al., 2017). The complete chloroplast genome sequences can be obtained from GenBank under the accession numbers PV995076 (*N. langsdorffii*) and PV995077 (*N. longiflora*). The chloroplast genome sequences of *N. tabacum*, *N. benthamiana*, *N. glauca*, and *N. otophora* were obtained from published sources (Asaf et al., 2016; Stegemann et al., 2012; Yukawa et al., 2005). The chloroplast genome sequences of different species were compared with Geneious 2024.0 (https://www.geneious.com).

### Construction of chloroplast transformation vectors

As targeting region for plastid transformation, the *psaB*/*trnS* region (Figure 2) from the plastid genomes of Nlong, Nlangs, Noto, Nglau, and Nbent was cloned as PCR products amplified with primers oQLU17 and oQLU18 (Table S1) between the Acc65I and EcoICRI restriction sites of vector pBluescript II SK (−). A chimeric *aadA* gene (Svab & Maliga, 1993) conferring resistance to spectinomycin (Spec) and streptomycin (Strep) and codon‐optimized for chloroplast expression was sourced from plasmid pDK308 (Agrawal et al., 2020; Strand et al., 2023). The *aadA* coding region was fused to the promoter of the *Chlamydomonas reinhardtii* plastid *psaA* gene and the 3´ UTR from the *C. reinhardtii* plastid *atpB* gene. The cassette was then amplified with primers oQLU19 and oQLU20 (Table S1) and inserted into the SpeI site located in the spacer between the *trnfM* and *trnG* genes (Figure 2). The resultant species‐specific vectors (pQL6, pQL7, pQL8, pQL9, and pQL10; Figure 2) are tailored for five different *Nicotiana* species: Nlong, Nlangs, Noto, Nglau, and Nbent.

A dsRed expression cassette was introduced into the plastid transformation vectors for visual tracking of transformation events. The cassette includes the *dsRed* gene, controlled by the plastid ribosomal RNA operon promoter and a chimeric 5' UTR derived from the *N. tabacum* chloroplast *psbA* and *clpP* genes (Hertle et al., 2021), and is part of a bicistronic operon together with the *aadA* gene (Figure 2). The operon was PCR amplified with primers oQLU33 and oQLU34 (Table S1) and inserted into the same integration site used in pQL6 and pQL10, generating the final plastid transformation vectors pQL11 and pQL15 for Nlong and Nbent, respectively (Figure 2). All vector sequences are available from GenBank (accession numbers: pQL6, PX281418; pQL7, PX281419; pQL8, PX281420; pQL9, PX281421; pQL10, PX281422; pQL11, PX281423; pQL15, PX281424), and the vectors can be obtained upon request.

### Biolistic transformation and selection of transplastomic lines

Chloroplast transformation experiments in Nbent, Nglau, Nlangs, Nlong, and Noto were carried out using a Bio‐Rad PDS‐1000/He biolistic gun equipped with a hepta adaptor. Young leaves of aseptically grown 5–6‐week‐old plants raised on agar‐solidified MSsuc2 medium were bombarded with plasmid DNA‐coated 0.6 μm gold particles (Bio‐Rad) following published protocols (Ruf & Bock, 2021). The bombarded leaves were cut into 5 × 5 mm pieces and cultured on SIM supplemented with spectinomycin. With the exception of Nlong, for which spectinomycin concentrations of 500, 1000, and 2000 mg L^−1^ were used for the selection of transplastomic clones, the explants of all other species underwent selection at a spectinomycin concentration of 500 mg L^−1^. For the different species, individually optimized SIMs (containing different cytokinins) were used, as detailed in Table 2. The cultured explants were transferred to fresh SIM every 5–8 weeks (depending on the species) to ensure maintenance of a constant hormone concentration and humidity in the selection plates.

Putative transplastomic shoots and calli exhibiting resistance to spectinomycin gradually emerged during primary selection, with the time frames of appearance of antibiotic‐resistant lines being variable between species (Table 2). Each independently obtained resistant line was assigned a unique number, and the resistant tissue was separated from the surrounding sensitive tissue for further individual cultivation. Resistant calli were continuously cultured on SIM supplemented with spectinomycin until shoot differentiation occurred. Tissue samples were then taken from leaves of regenerated antibiotic‐resistant shoots and subjected to additional regeneration cycles on the species‐specific SIM with spectinomycin. In addition, leaf explants were subjected to resistance tests in the presence of streptomycin (500 mg L^−1^). Spontaneous spectinomycin‐resistant mutants that can appear upon primary selection typically exhibit sensitivity to streptomycin, which distinguishes them from transplastomic lines that are resistant to both aminoglycoside antibiotics (Bock, 2001). Once a putative transplastomic line had been confirmed as resistant to both spectinomycin and streptomycin, shoots regenerated from the spectinomycin‐containing SIM in the additional selection rounds were transferred to Magenta boxes containing MSsuc2 medium supplemented with 500 mg L^−1^ spectinomycin for further growth and rooting. In some species, it was observed that the efficiency of rooting was reduced by excessive humidity in the culture vessel, and therefore, sealing of the vessels (Magenta boxes) with tape was avoided. In addition, plant growth was carefully monitored, and downward bending leaves were cut back to prevent loss of medium contact of the stem base. After successful rooting, samples of leaf tissue were harvested for RFLP analysis, and confirmed homoplasmic transplastomic plants were transferred to the greenhouse for growth to maturity and seed production.

### Genetic crosses and inheritance assays

Flowering transplastomic *Nicotiana* plants were subjected to both self‐pollination and cross‐pollination with WT plants to verify maternal inheritance of the engineered plastids. In Noto, self‐pollination is very inefficient due to the physical separation between stigma and anthers. In the natural habitat of the species, pollination is facilitated by bats (Tiedge & Lohaus, 2017), and manual intervention was necessary to perform both self‐pollination and cross‐pollination in the greenhouse setting. While artificial self‐pollination was not a requirement in the other four *Nicotiana* species, it was employed to increase seed yields.

Seeds were collected from capsules originating from self‐pollinated and cross‐pollinated flowers and germinated on MSsuc2 medium supplemented with 500 mg L^−1^ spectinomycin. Seedlings harboring transgenic plastids are green in the presence of the antibiotic, whereas seedlings with WT plastids bleach out, thus allowing determination of the inheritance patterns of the transgenic chloroplast genomes.

### Isolation of nucleic acids and DNA gel blot analyses

Total genomic DNA was extracted from fresh leaf tissue employing a CTAB‐based protocol (Doyle & Doyle, 1990). DNA samples were digested with the restriction enzyme BglII, and the fragments were separated by electrophoresis in 1% (w/v) agarose gels. The DNA fragments were then transferred onto Hybond‐XL nylon membranes (GE Healthcare) using capillary blotting. For Southern blot analysis, a 541 bp probe corresponding to the *psaB* gene from *N. tabacum* was used. The probe was generated by PCR with primers oQLU36 and oQLU37 (Table S1), and the resulting amplification product was purified by agarose gel electrophoresis and extracted from excised gel slices using the NucleoSpin Extract II kit (Macherey‐Nagel). Probe labeling was achieved by incorporating α[^32^P]dCTP using the Multiprime DNA Labeling System (GE Healthcare) based on random priming. Hybridization was conducted in Rapid‐Hyb buffer at a temperature of 65°C, following the manufacturer's instructions. After appropriate washing steps to remove non‐specifically bound probe molecules, the membranes were exposed to phosphorimaging screens, followed by scanning in a Typhoon TRIO+ scanner (GE Healthcare).

### Confocal laser‐scanning microscopy

Subcellular localization of chlorophyll fluorescence and the transgenically expressed dsRed protein were assessed using a Leica TCS SP8 confocal laser‐scanning microscope. DsRed protein fluorescence was excited with a 561 nm diode‐pumped solid‐state laser, and emission was detected between 575 and 603 nm. Chlorophyll fluorescence was excited using the same diode laser, and emission was detected with a 650 to 750 nm filter.

## AUTHOR CONTRIBUTIONS

QL, SR, and RB designed the research. QL performed most experiments. SO participated in transformation and chloroplast isolation experiments. SC conducted the bioinformatic analysis of genome sequencing data. All authors participated in data evaluation. RB wrote the manuscript, with input from all co‐authors.

## CONFLICT OF INTEREST

The authors declare no competing interests.

## Supporting information


**Figure S1.** Evaluation of the *in vitro* regeneration capacity of *N. acumiata*, *N. alata*, *N. attenuata*, and *N. glauca* explants.
**Figure S2.** Evaluation of the *in vitro* regeneration capacity of *N. glutinosa*, *N. kawakamii*, *N. langsdorffii*, and *N. longiflora* explants.
**Figure S3.** Evaluation of the *in vitro* regeneration capacity of *N. noctiflora*, *N. obtusifolia*, *N. paniculata*, and *N. sylvestris* explants.
**Figure S4.** Evaluation of the regeneration capacity of *N. tabacum*, *N. tomentosiformis*, and *N. undulata*.
**Figure S5.** Test of *N. longiflora* leaf explants for sensitivity to spectinomycin and streptomycin.
**Figure S6.** DNA sequence comparison of the genomic region from *psaB* to *psbC* of six species in the genus *Nicotiana*.
**Figure S7.** RFLP analysis of Nlong‐QL11 transplastomic lines.
**Table S1.** List of oligonucleotides used in this study.

## Data Availability

Data supporting the findings of this work is available within the manuscript and its associated Supplementary Information.
