# Supplementary material for: Development of chloroplast transformation for five species in the genus Nicotiana
Source: Plant J. 2025 Oct 27;124(2):e70542. doi: 10.1111/tpj.70542 (PMC12558618; doi:10.1111/tpj.70542)
Supplement: Supplementary file 1 — Figure S1. Evaluation of the in vitro regeneration capacity of N. acumiata, N. alata, N. attenuata, and N. glauca explants. Figure S2. Evaluation of the in vitro regeneration capacity of N. glutinosa, N. kawakamii, N. langsdorffii, and N. longiflora explants. Figure S3. Evaluation of the in vitro regeneration capacity of N. noctiflora, N. obtusifolia, N. paniculata, and N. sylvestris explants. Figure S4. Evaluation of the regeneration capacity of N. tabacum, N. tomentosiformis, and N. undulata. Figure S5. Test of N. longiflora leaf explants for sensitivity to spectinomycin and streptomycin. Figure S6. DNA sequence comparison of the genomic region from psaB to psbC of six species in the genus Nicotiana. Figure S7. RFLP analysis of Nlong‐QL11 transplastomic lines. Table S1. List of oligonucleotides used in this study. [file TPJ-124-0-s001.pdf]

SUPPLEMENTARY INFORMATION

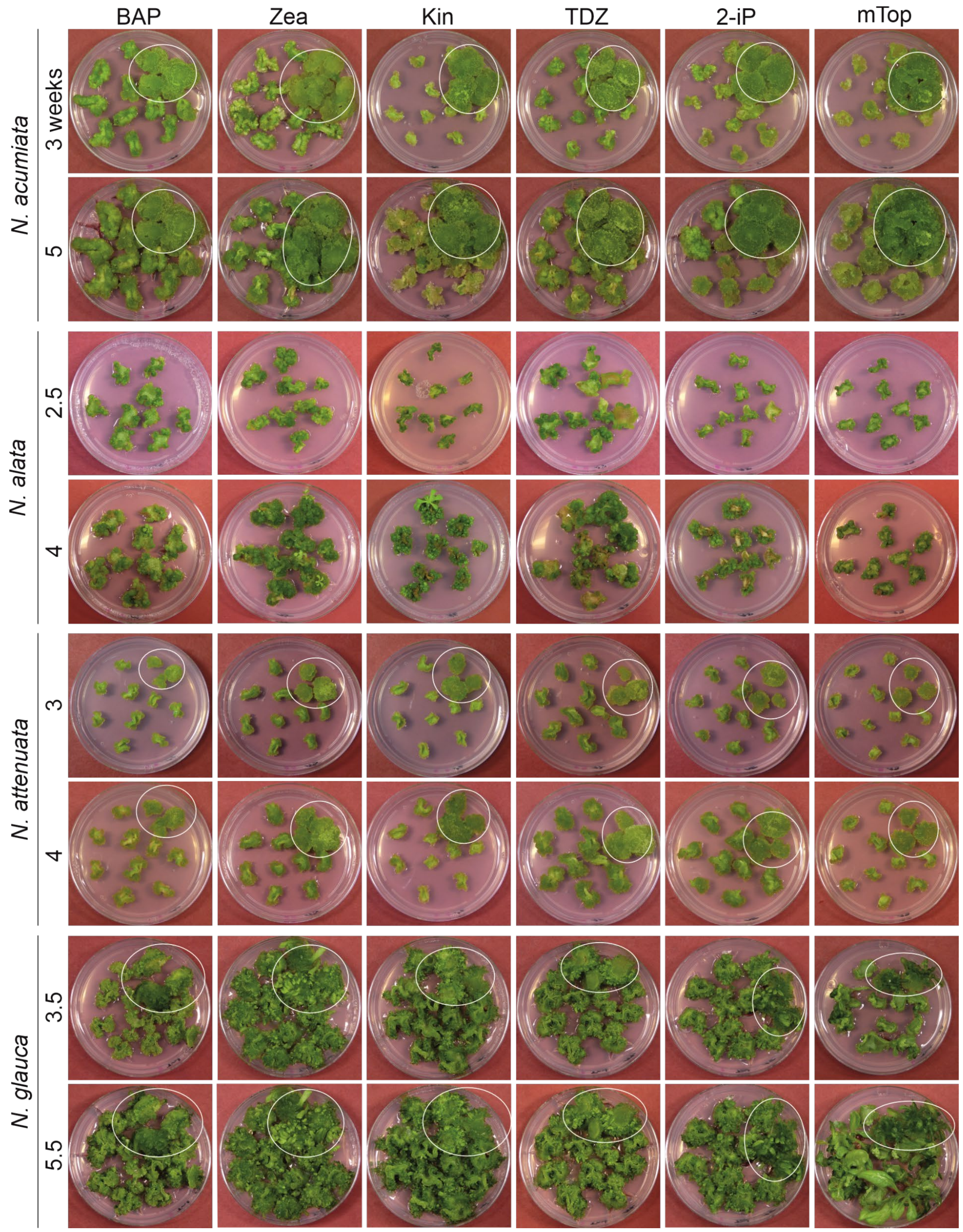

Fig. S1. Evaluation of the *in vitro* regeneration capacity of *N. acumiata*, *N. alata*, *N. attenuata*, and *N. glauca* explants. Regeneration media with six different cytokinins were tested in this experiment, and regeneration was documented at two time points.

Stem discs tested for regeneration are located within the white circle in the upper right corner of the Petri dishes. All other explants were from leaves. Stem discs of *N. alata* were not tested. BAP: 6-benzylaminopurine; Kin: kinetin; mTOP: meta-topolin; TDZ: thidiazuron; Zea: zeatin; 2-iP: N6-2-isopentenyladenine.

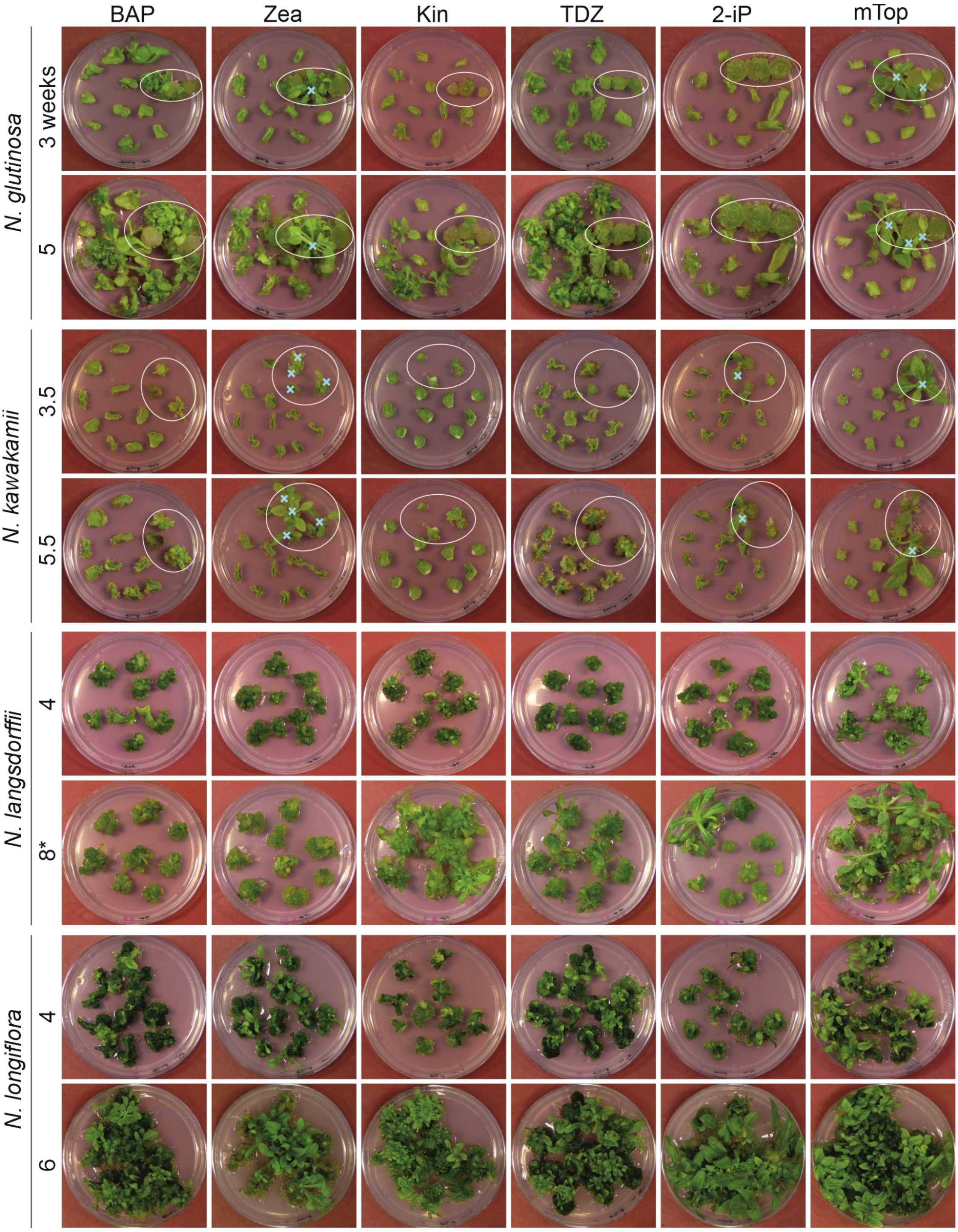

Fig. S2. Evaluation of the *in vitro* regeneration capacity of *N. glutinosa*, *N. kawakamii*, *N. langsdorffii,* and *N. longiflora* explants. The light blue crosses mark shoots that likely did not originate from regeneration, but rather from axillary bud outgrowth from stem explants. Stem discs of *N. langsdorffii* and *N. longiflora* were not tested. The medium was changed once (after six weeks) for *N. langsdorffii*, because the

explants of this species showed a slower response than those of the other species. For details and abbreviations of hormones, see Suppl. Figure 1.

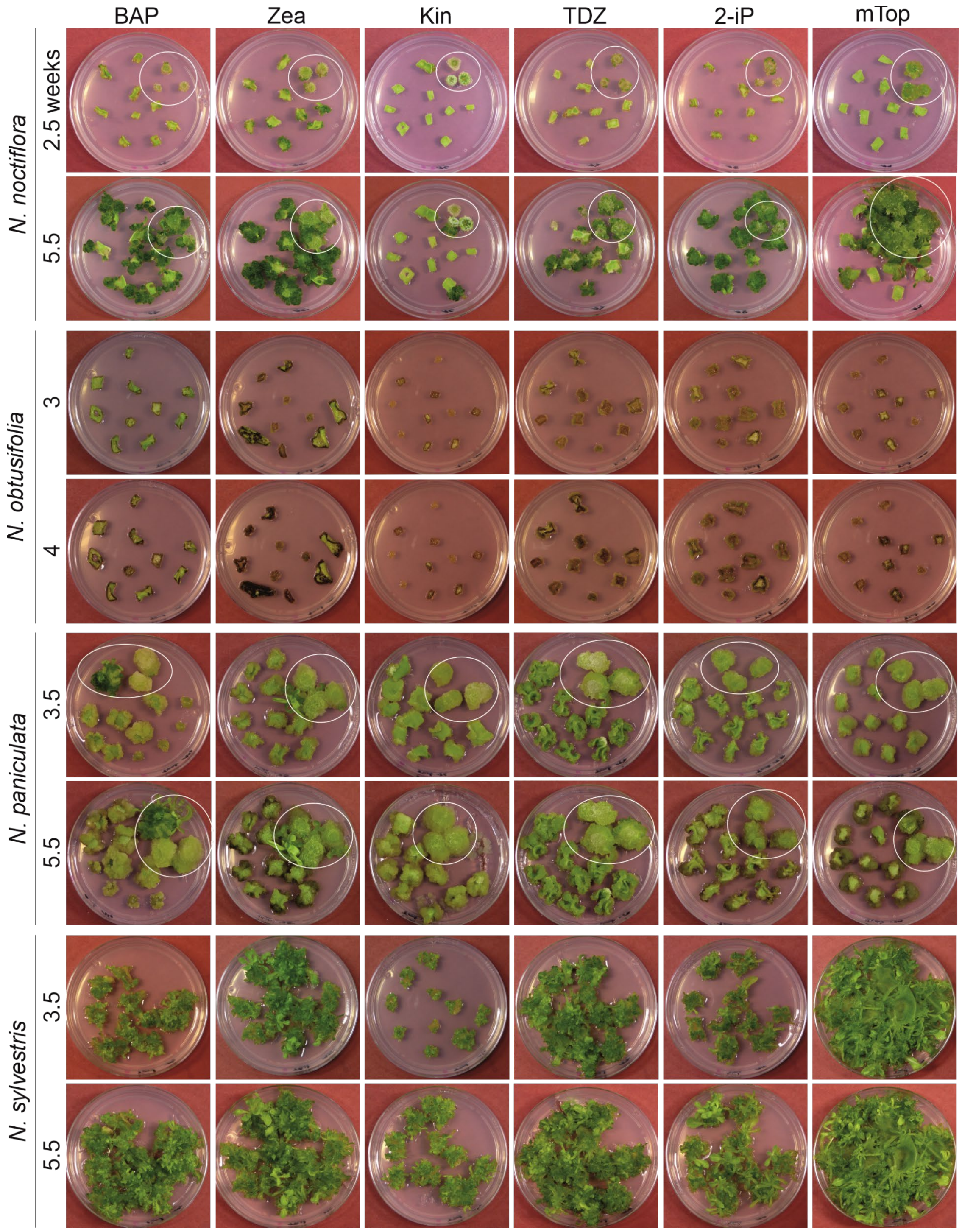

Fig. S3. Evaluation of the *in vitro* regeneration capacity of *N. noctiflora*, *N. obtusifolia*, *N. paniculata,* and *N. sylvestris* explants. Stem discs of *N. obtusifolia* and *N. sylvestris* were not tested. For details and abbreviations of hormones, see Suppl. Figure 1.

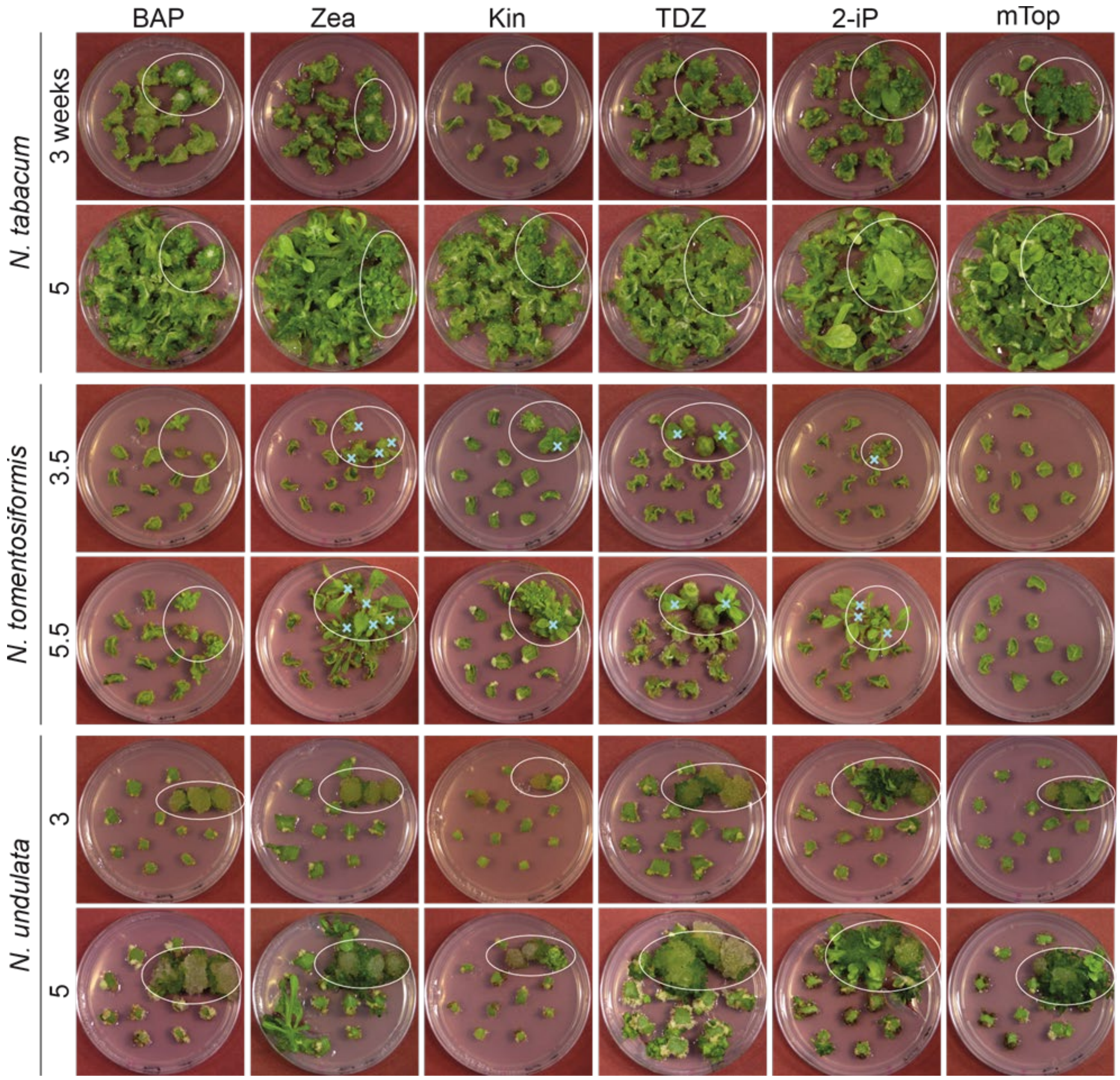

Fig. S4. Evaluation of the regeneration capacity of *N. tabacum*, *N. tomentosiformis*, and *N. undulata*. The light blue crosses mark shoots that likely did not originate from regeneration, but rather from axillary bud outgrowth from stem explants. For details and abbreviations of hormones, see Suppl. Figure 1.

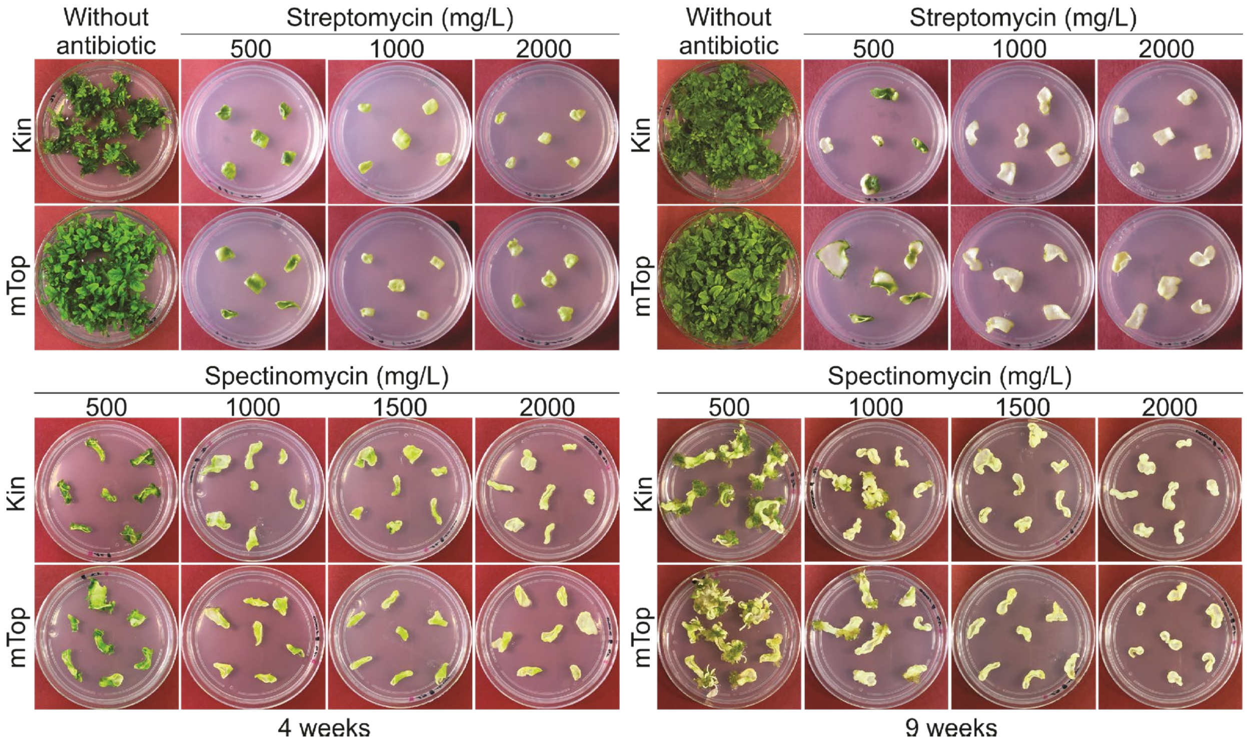

Fig. S5. Test of *N. longiflora* leaf explants for sensitivity to spectinomycin and streptomycin. The images show the regeneration response of Nlong wild-type leaf pieces on media with kinetin (Kin) or meta-topolin (mTOP) as cytokinin, and different concentrations of spectinomycin or streptomycin. Sensitivity was assessed after incubation for 4 weeks and 9 weeks.

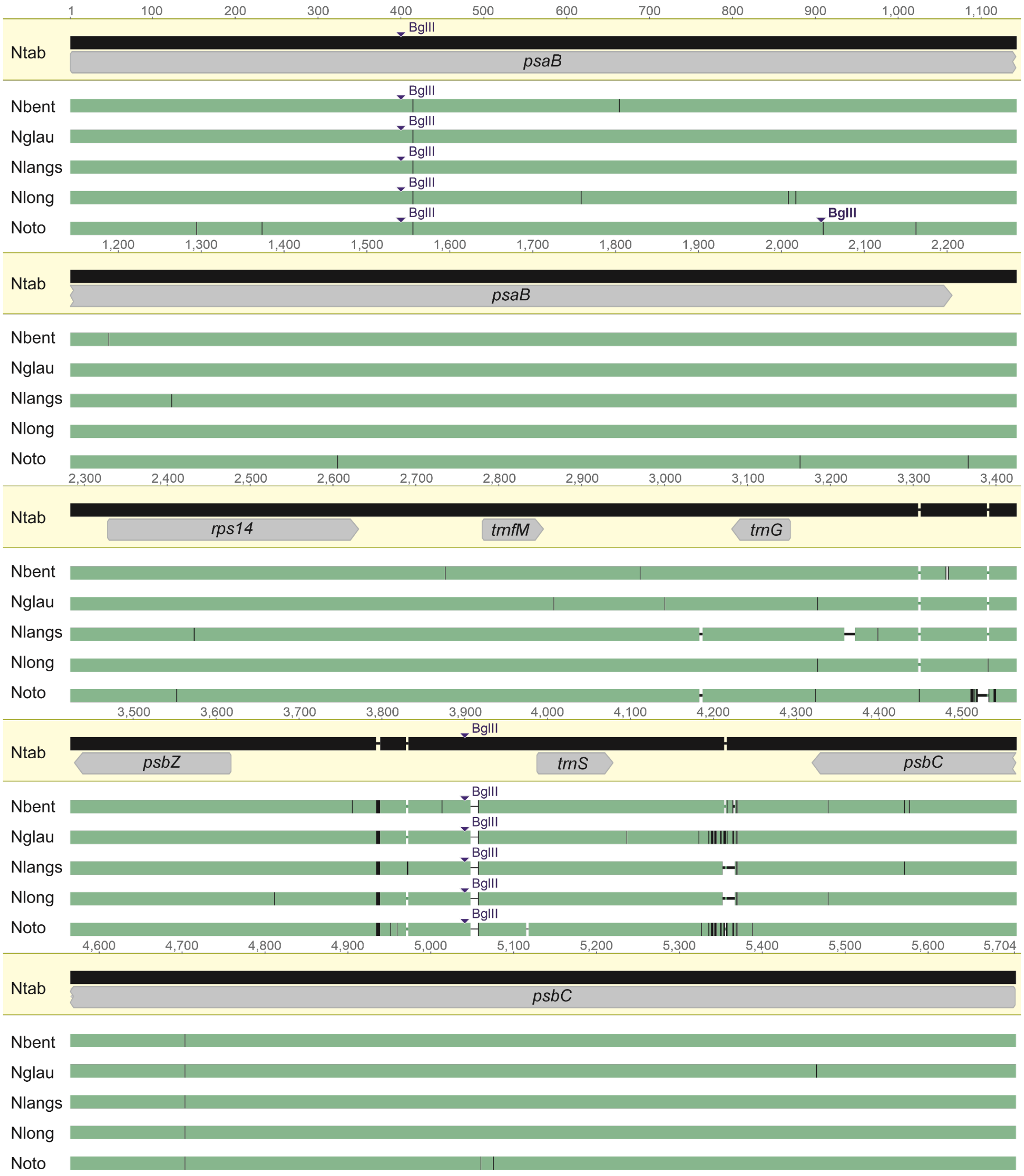

Fig. S6. DNA sequence comparison of the genomic region from *psaB* to *psbC* of six species in the genus *Nicotiana*. The size of the aligned region is approximately 5.8 kb, and genes contained in this region are indicated as grey bars with the arrows indicating the direction of transcription (cf. Figure 2). The DNA sequence of the reference species Ntab is represented as black bar, and the DNA sequences of the five *Nicotiana* species used for the development of plastid transformation protocols in this study (Nbent, Nglau, Nlangs, Nlong, Noto) are represented in green. Single-nucleotide

polymorphisms (SNPs) compared to Ntab are indicated by black vertical lines, with thicker lines denoting multiple consecutive nucleotide polymorphisms. Deletions relative to Ntab are marked by horizontal black lines. Horizontal black lines in the Ntab sequence indicate insertions present in other *Nicotiana* species. When only one or two species carry a particular insertion (while the other species lack it and are similar to Ntab), the insert is indicated by a green horizontal line in the species that are similar to Ntab. The BglII restriction sites are labelled, and an additional BglII site present within the *psaB* coding region of the species Noto is shown in bold.

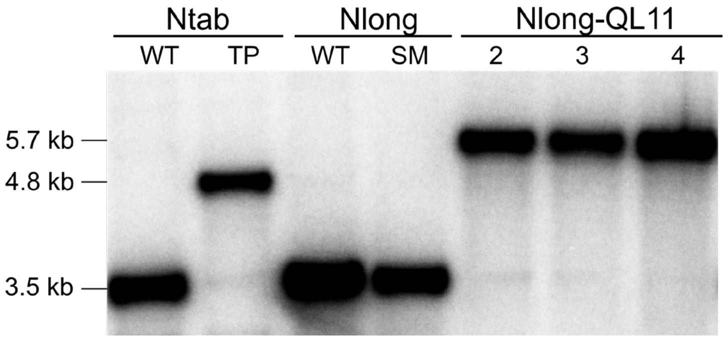

Fig. S7. RFLP analysis of Nlong-QL11 transplastomic lines. WT: wild type; TP: transplastomic plant; SM: spontaneous spectinomycin-resistant mutant. For further details, see Figure 4.

Table S1. List of oligonucleotides used in this study. F: forward, R: reverse.

Use	Name	Sequence (5' → 3')	Length of PCR product (bp)
Cloning: amplification of the *psaB/ trnS* region from wild-type plants	oQLU17 F<br>oQLU18 R	GGGCGAATTGGGTACCAACCGCTCAGCCATCTCTC<br>GAACAAAAGCTGGAGCTCTTCACAACTTATCAACGG	Nbent: 2144<br>Nglau: 2144<br>Nlong: 2145<br>Nlangs: 2129<br>Noto: 2128
Cloning: amplification of the *aadA* cassette from a transplastomic *N. tabacum* plant generated with vector pDK308	oQLU19 F<br>oQLU20 R	GGAATTAATTACTAGTTTTAACACATTATTAAATAC<br>GGTTGACTCTGTTAACTAGCTTTTTACGAATACACATAT	1238
Cloning: amplification of the *dsRed::aadA* cassette from a transplastomic *N. tabacum* plant generated (Hertle et al., 2021)	oQLU33 F<br>oQLU34 R	GGAATTAATTACTAGCGGCCGCTCTAGCTAGAACT<br>GACTCTGTTAACTAGCGCCGTCGTTCAATGAGAAT	2213
Hybridization probe derived from the *psaB* gene of *N. tabacum*	oQLU36 F<br>oQLU37 R	CGTGCATCTAAAGCACCTTT<br>GCACAAGACTTTACTACTCA	541
